# *Tetraselmis chuii* as Source of Bioactive Compounds Against *Helicobacter pylori*: An Integrated Proteomic and Bioactivity Approach

**DOI:** 10.3390/molecules30244669

**Published:** 2025-12-05

**Authors:** Marta Majchrzak, Samuel Paterson, Pilar Gómez-Cortés, Jose Manuel Silvan, Adolfo J. Martinez-Rodriguez, Blanca Hernández-Ledesma

**Affiliations:** 1 Institute of Food Science Research (CIAL, CSIC-UAM, CEI UAM + CSIC), Nicolás Cabrera 9, 28049 Madrid, Spain; marta.majchrzak@csic.es (M.M.); samuel.paterson@csic.es (S.P.); p.g.cortes@csic.es (P.G.-C.); jm.silvan@csic.es (J.M.S.); 2 Department of Applied Physical Chemistry, Autonoma University of Madrid, Francisco Tomás and Valiente, 7, 28049 Madrid, Spain; 3 Department of Nutrition and Food Science, Complutense University of Madrid, Plaza de Ramón y Cajal, s/n, 28040 Madrid, Spain

**Keywords:** microalgae, *Tetraselmis chuii*, bioactive peptides, in silico digestion, *Helicobacter pylori*

## Abstract

Microalgae represent sustainable sources of bioactive compounds with potential health benefits. This study conducted a comprehensive proteomic analysis of *Tetraselmis chuii* (*T. chuii*) biomass to identify proteins capable of generating bioactive peptides (BAPs) through simulated orogastric digestion. In silico digestion and bioinformatic predictions indicated the release of antioxidant, anti-inflammatory, and antibacterial peptides. Molecular docking demonstrated strong interactions with targets implicated in oxidative stress, inflammation, and *Helicobacter pylori* (*H. pylori*) virulence. Biochemical assays and cell-based models confirmed antioxidant and anti-inflammatory activities in both the biomass and its digest, although no significant antibacterial effect against *H. pylori* was observed under the tested conditions. Considering the role of chronic inflammation in *H. pylori*-associated pathologies, these findings suggest that *T. chuii* may serve as a candidate for mitigating tissue damage driven by oxidative and inflammatory stress. Further research is required to address compound stability and optimize delivery strategies.

## 1. Introduction

Microalgae are eukaryotic, photosynthetic, single-celled microorganisms that thrive in aquatic environments [1]. Beyond their role as a sustainable source of high-quality proteins, microalgae produce bioactive compounds such as omega-3 fatty acids (PUFAs), phytosterols, vitamins, carotenoids (e.g., lutein, astaxanthin, and β-carotene), and bioactive peptides (BAPs) [2]. These compounds exhibit health-promoting properties, including antioxidant, antibacterial, antihypertensive, immunomodulatory, and anticancer effects [3,4]. The genus *Tetraselmis*, belonging to the family *Chlorodendraceae*, includes a diverse group of flagellate and motile unicellular microalgae, green in color due to the presence of chlorophyll a and b. Their spherical cells feature an invagination with four identical flagella arranged in two opposing pairs [3].

The antioxidant properties of microalgae are linked to their bioactive compounds, which can neutralize free radicals in the body, reducing their harmful effects and helping to mitigate aging and oxidative stress-associated chronic diseases [5]. Compared to other antioxidant compounds in microalgae, BAPs exhibit superior stability, higher bioavailability, and multifunctional activity, enhancing their efficacy against oxidative stress and overall health benefits [6]. The antioxidant potential of BAPs is influenced by factors such as chain length and the specific composition of amino acids (AAs), especially those with outstanding free radical-scavenging capabilities. Among the various types of BAPs, antibacterial peptides are notable for their composition, generally ranging from 12 to 50 AAs [7]. These peptides are synthesized by a wide array of organisms and cells, playing a fundamental role in host defense mechanisms against pathogens as part of the innate immune system [8].

Although limited, existing evidence highlights studies that have characterized the anti-inflammatory and antioxidant properties of BAPs from microalgae. Vo and Kim (2013) demonstrated that two peptide sequences (LDAVNR and MMLDF), released from *Spirulina maxima* proteins via enzymatic hydrolysis, inhibited inflammation by reducing histamine release, interleukin-8 (IL-8) production, and reactive oxygen species (ROS) generation in histamine-stimulated RBL-2H3 mast cells and EA.hy926 endothelial cells [9]. In the study, carried out by Fan et al. [10], the antioxidant peptide VECYGPNRPQF was purified from the hydrolysate of *Chlorella vulgaris* with pepsin, while Guzmán et al. [11] identified de novo sequences of antibacterial peptide LWFYTMW in the microalgae *T. suecica*. However, limited information regarding microalgae proteins that could serve as precursors of BAPs restricts our understanding of the specific peptide sequences responsible for the observed biological effects [12].

Current technologies for obtaining BAPs include direct extraction from tissues, chemical synthesis, and recombinant DNA techniques [13]. However, due to their high cost, alternative approaches such as enzymatic hydrolysis and microbial fermentation have emerged as more economically viable and promising alternatives [14]. Moreover, BAPs can be released in vivo during the gastrointestinal digestion process [15]. Nevertheless, current understanding of BAPs generation from microalgae during digestion and their subsequent bioavailability remains limited [16]. Given the limitations of in vitro models, recent years have seen increasing adoption of in silico digestion models that leverage existing experimental data [17]. In silico gastrointestinal digestion consists of the enzymatic cleavage of target proteins, using pepsin, trypsin, and chymotrypsin as digestive enzymes, and using bioinformatics tools such as UniProt Knowledgebase (UniProtKB), BIOPEP-UWM, ExPAS, PeptideCutter, and MS-Digest. After in silico digestion, the resulting peptide sequences undergo further analysis for BAPs screening, such as PeptideRanker and BIOPEP-UWM [18]. In silico models have been used to evaluate the bioactivity of peptides from proteins of various marine species, such as microalgae. In a study carried out by Hayes et al. [19], the potential of certain peptides from the digestion of *Nannochloropsis oculata* to act as Angiotensin-converting enzyme (ACE) inhibitors was demonstrated. In another study carried out by Xie et al. [20], ACE inhibitory peptides from *C. vulgaris* were determined after in silico gastrointestinal digestion of its proteins. However, to our knowledge, there is a lack of evidence on the antioxidant, anti-inflammatory, and antibacterial properties of *T. chuii* biomass after being subjected to in silico and in vitro orogastric digestion.

*Helicobacter pylori* (*H. pylori*) is one of the most common pathogenic bacteria in humans, infecting over 50% of the world’s population. *H. pylori* infection induces oxidative stress and chronic inflammation, which are linked to gastric cancer, making it the most potent known risk factor associated with *H. pylori* infection [8]. *H. pylori* colonization of gastric epithelial cells triggers a robust inflammatory response characterized by neutrophil and macrophage infiltration, leading to ROS production in host tissues [12]. Consequently, therapeutic strategies targeting inflammatory and oxidative pathways in gastric epithelium have demonstrated significant efficacy in managing *H. pylori*-related pathology [13]. Current eradication therapy combines antibiotics with potent acid suppressants to improve efficacy and mitigate side effects [21]. However, the increasing prevalence of antibiotic-resistant strains has prompted the search for novel antibacterial, antioxidant, and/or anti-inflammatory compounds to better manage this infection [8].

Notably, peptides demonstrating effectiveness against *H. pylori* are derived from both natural and synthetic origins [8]. Moreover, beyond the antibacterial category, other BAPs, particularly those exhibiting anti-inflammatory and antioxidant properties, hold substantial promise for the therapeutic management of *H. pylori*-associated conditions [22]. The novelty of our study lies in the implementation of an integrative approach to evaluate the bioactive potential of *Tetraselmis chuii*, combining in vitro simulated gastrointestinal digestion (INFOGEST 2.0), advanced peptide analysis, and functional validation in human gastric epithelial cells (AGS). Unlike previous studies, which have mainly focused on compositional characterization or isolated biochemical assays, our strategy enables a direct correlation between the post-digestive peptide profile and the bioactive effects observed at the cellular level. This approach acknowledges that certain peptides generated during digestion can retain significant biological activity, even under simulated physiological conditions. This study aimed to explore the proteome of *T. chuii* microalgae as a source of BAPs. The objective was first to identify candidate proteins through proteomic analysis and then to predict their bioactivity via in silico digestion and molecular docking. These predictions were subsequently validated experimentally by testing the antioxidant, anti-inflammatory, and anti-*H. pylori* effects of the in vitro orogastric digest in cell culture.

## 2. Results and Discussion

### 2.1. Characterization of T. chuii Proteins

In order to characterize the protein profile of *T. chuii* biomass, an initial measurement of the protein content was carried out by the BCA assay, obtaining a value of 32.29 ± 1.62% of protein. This value was similar to that measured by the Kjeldahl method, 32.19 ± 0.29% [23], indicating that the conversion factor used (5.95) was adequate to convert the nitrogen content of this microalgae into protein. When the microalgae underwent orogastric digestion, the protein concentration in the digest decreased to a protein value of 28.04 ± 2.05%, likely due to protein degradation and the release of free AAs.

**Figure 1:**
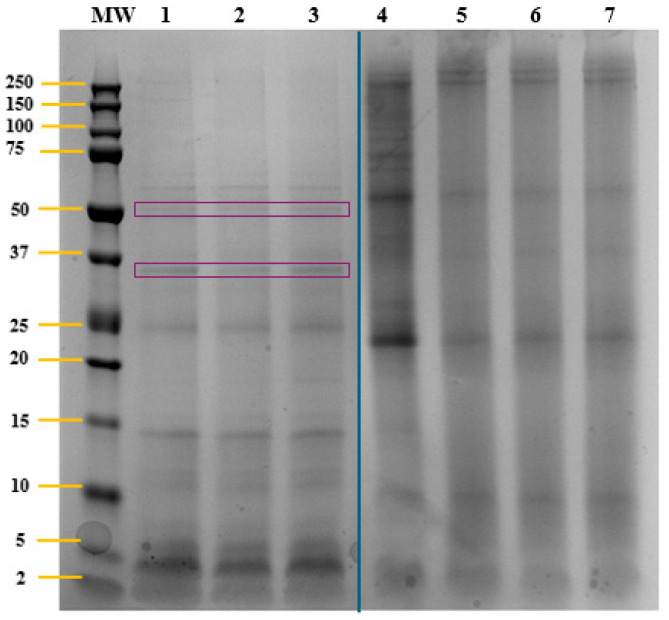
Electrophoresis gel (SDS-PAGE) of *Tetraselmis chuii* biomass and orogastric digests (50 μg protein/mL). MW: protein molecular weight marker (2–250 kDa); lines 1–3: digestion control; line 4: biomass; lines 5–7: orogastric digest. Lanes 1–3 and 4–7 originate from the same gel but were rearranged by splicing after removing four lanes. The dividing line indicates the splice junction. No adjustments were made that could alter the interpretation of the results. Purple grid lines correspond to pepsin (38.3 kDa) and gastric lipase (52 kDa) bands.

The protein profile of the biomass and orogastric digests of *T. chuii* was analyzed by SDS-PAGE (Figure 1). First, in the digestion control (Lines 1–3), the bands corresponding to the gastric enzymes used, pepsin (38.3 kDa) and gastric lipase (52 kDa) [24] were observed. In addition, low MW bands (2–75 kDa) could correspond to proteins and peptides contained in saliva, such as salivary α-amylase, whose MW is approximately 56 kDa [25]. Moreover, in *T. chuii* biomass (Line 4), bands with MW ranging from 5 to 250 kDa were observed. Some of these bands could correspond to chloroplast-related proteins, which have been previously identified, with MW between 7.8 and 46.0 kDa [26]. Electrophoretic analysis indicated partial digestion of *T. chuii* proteins after the action of salivary and gastric enzymes, with reduced band intensity compared to undigested biomass and the complete disappearance of some bands (Lines 5–7).

Following the in-gel digestion and subsequent RP-LC-MS/MS analysis of *T. chuii* biomass, PEAKS and SPIDER search tools [27] were utilized to identify a total of 4184 peptide-spectrum matches (PSMs). The results included 4135 peptide-spectrum matches from MS/MS scans, along with 2110 features found only in database searches. Additionally, 2164 modified peptide sequences (excluding isoleucine (I)/leucine (L) differentiation) and 1764 unmodified features without I/L distinction were identified. Since sequences are essential for protein identification based on homology, PEAKS clusters proteins into groups containing all those identified by the same set of peptides [28]. In this study, 332 proteins were detected through PEAKS Studio v11.5 search engine. Of them, 323 proteins were identified using two or more unique peptides, and 314 correspond to groups of proteins. The corresponding accession numbers, −10logP values, peptide counts (both total and unique), the types of peptide modifications (PTMs), average masses, and descriptions of all detected proteins in *T. chuii* biomass are compiled in Supplementary Table S1. The number of proteins identified in this study was lower than that reported for other microalgae [29], reflecting the incomplete *T. chuii* proteome data currently available in databases. Despite this limitation, our work provides the first characterization of proteins in the *T. chuii* biomass. Future comprehensive mapping of the *T. chuii* proteome will be crucial to uncover factors affecting its proteins and evaluate their potential as a source of BAPs. The anti-inflammatory potential of microalgal proteins is increasingly recognized, although most studies have focused on peptide hydrolysates rather than fully characterized intact proteins. Notably, prolyl endopeptidase was detected and has been associated with the degradation of pro-inflammatory neuropeptides such as substance P and bradykinin, thereby modulating nuclear factor (NF)-κB signaling and reducing tumor necrosis factor (TNF)-α and IL-6 expression [30,31]. Similarly, a SUEL-type lectin domain-containing protein was identified, which may interact with glycan structures on immune cells and attenuate macrophage activation and cytokine release [32]. In *Arthrospira platensis*, the biliprotein C-phycocyanin has been extensively characterized for its ability to inhibit cyclooxygenase (COX)-2, inducible nitric oxide synthase (iNOS), and pro-inflammatory cytokines via suppression of NF-κB and MAPK pathways [33,34]. In *T. suecica*, lectin-like proteins have been proposed to modulate immune responses through glycan recognition, although their molecular targets remain to be fully elucidated [32]. Additionally, aconitate hydratase, identified in the current dataset, has been implicated in redox-sensitive regulation of inflammatory responses, acting as a metabolic sensor that influences NF-κB-dependent transcription under oxidative stress [35].

**Figure 2:**
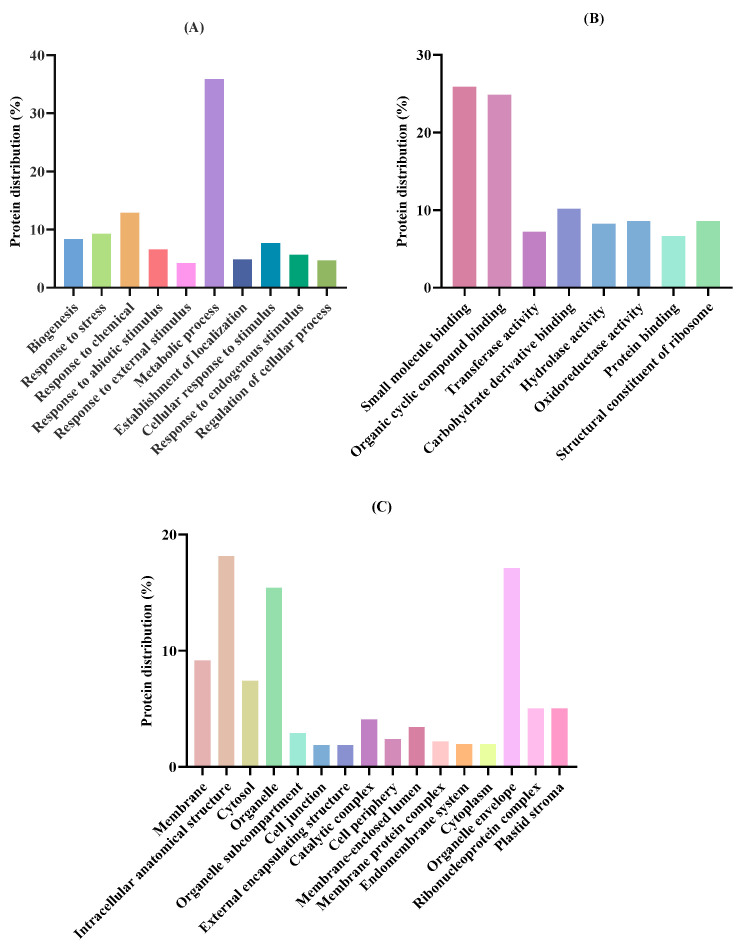
Proteomic functional distribution of identified proteins from *Tetraselmis chuii* biomass using gene ontology (GO): (**A**) Biological process GO level 3 distribution; (**B**) Molecular function GO level 3 distribution; (**C**) Cellular component GO level 3 distribution. Data are represented in percentages.

To clarify the function of the 323 proteins identified in *T. chuii*, protein sequence alignments were performed using the OmicsBox 3.3.2 bioinformatic software through the BLAST2GO methodology. Protein functions were annotated according to the Gene Ontology (GO) framework, which comprises three main categories: cellular component, molecular function, and biological process. The distribution of proteins across levels 1 and 3 for each functional group is shown in Supplementary Figure S1, while the complete distribution for all levels is detailed in Supplementary Figures S2–S4. In general terms, the distribution of GO annotations in *T. chuii* was homogeneous, as the proportions associated with each GO category were similar: 259 annotations (32.87%) corresponded to biological processes, 266 (33.76%) to molecular functions, and 263 (33.38%) to cellular components. Regarding the classification of the group of biological processes (Figure 2A), these were subclassified into various processes, highlighting metabolic processes (35.89%), followed by response to chemicals (12.85%) and response to stress (9.25%). As for molecular functions (Figure 2B), the binding of small molecules (25.87%) and organic cyclic compounds (24.83%) stood out, with the minority being protein binding (6.64%). Finally, concerning cellular components (Figure 2C), proteins associated with the intracellular anatomical structure (18.12%), organelle envelope (17.1%), and organelles (15.43%) were the most predominant.

### 2.2. Effect of In Silico Gastric Digestion on Peptide Release

In a study carried out by Ji et al. [36], a protein–protein interaction network was identified in the marine microalgae *T. subcordiformis*, which provided a basis for the analysis of its metabolism. In this line, it is important to consider the interactions between proteins and other components of microalgae, which allow progress in the knowledge of the metabolic pathways involved. The proteomic analysis of *T. chuii* provides a comprehensive, functional perspective on the biological potential of this microalgae, focusing on the identification of BAPs released following simulated orogastric digestion. This methodological approach offers a more realistic insight into protein behavior under physiological conditions, assessing their stability and functionality after passage through the digestive system.

An in silico gastric digestion of the complete set of 323 identified sequences was performed to evaluate the potential peptides released by the action of pepsin on *T. chuii* proteins. Initial analysis identified 27,399 sequences, of which 11,023 corresponded to peptides. Following quantification of peptides released per protein, proteins yielding ≥ 75 peptides/protein were selected for further analysis. This subset, comprising 21 proteins, collectively generated 2083 peptides (Table 1). The average mass of the selected proteins ranged between 66 and 179 kDa. Among them, proteins that generated more peptides during simulated gastric digestion were the SUEL-type lectin domain-containing protein (171 peptides), followed by magnesium chelatase (146 peptides), and the pyruvate phosphate dikinase AMP/ATP-binding domain-containing protein (120 peptides). These proteins have been previously identified in other microalgae such as *C. vulgaris*, *Chlamydomonas reinhardtii*, and *A. platensis* [37,38,39].

**Figure 3:**
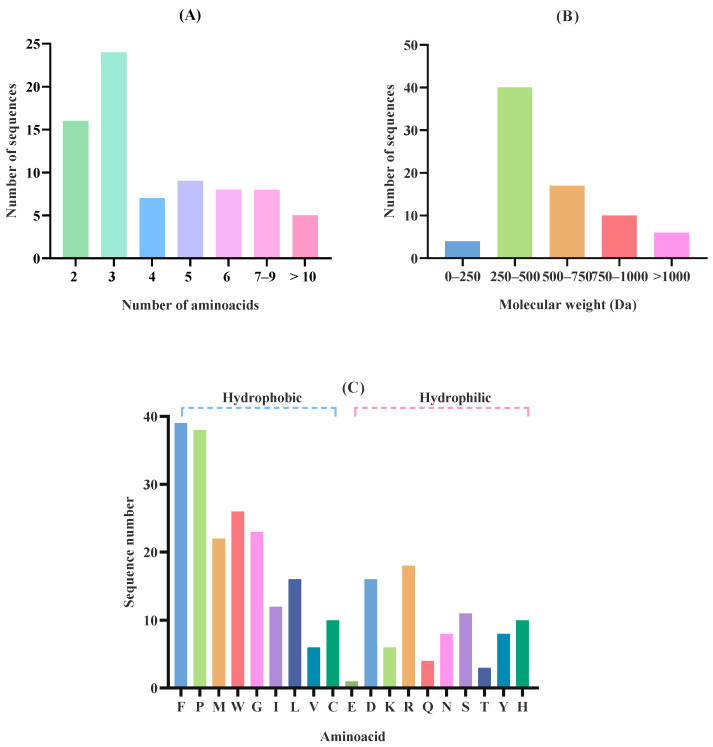
Protein profile of the peptide released after in silico gastric digestion from *Tetraselmis chuii* biomass: (**A**) Size distribution expressed as number of amino acids; (**B**) Molecular weight (MW) distribution (Da); (**C**) Amino acid distribution.

Selected (2083) peptides were classified according to their bioactivity by the Peptide Ranker software http://distilldeep.ucd.ie/PeptideRanker/ (accessed on 3 September 2024). Those peptides with a score ≥ 0.8 (77 peptides) were considered potentially bioactive. Most of the BAPs released after in silico gastric digestion were dipeptides (16) and tripeptides (24) (Figure 3A). Regarding the MW distribution (Figure 3B), peptides in the 250–500 Da range (40 peptides) and the 500–750 Da range (17 peptides) were the most prominent. Low-MW peptides, in general, have been associated with higher antioxidant capacity compared to high-MW peptides [40]. In addition, due to their small size, these peptides have a higher chance of crossing the intestinal barrier and reaching target organs to perform biological functions [41]. The main AAs were phenylalanine (F), proline (P) and tryptophan (W) (Figure 3C). Some AAs, such as tyrosine (Y), tryptophan (W), methionine (M), lysine (K), cysteine (C), and histidine (H), have been demonstrated to contribute to the antioxidant activity of peptides. While many of these exert direct antioxidant effects through electron donation and free radical neutralization, AAs like histidine (H) and lysine (K) can also exert indirect antioxidant effects by chelating pro-oxidant metal ions, thereby limiting metal-catalyzed oxidative reactions [42]. The neutralization capacity of ABTS radicals is attributed to the indole and benzene rings present in aromatic AAs [43]. In addition, the presence of aromatic rings in the molecule allows for charge stabilization, making ROS stable during the radical scavenging process [44].

Furthermore, certain BAPs with antibacterial properties against *H. pylori* constitute a chemically heterogeneous family that shares certain characteristics such as intermediate size (10–50 AAs), MW variable between 1 and 5 kDa, and an amphipathic character [45]. On the other hand, evidence confirms that plant-derived BAPs with an MW of about 500 Da and a chain length of between 2 and 6 AAs possess strong anti-inflammatory activity [46].

Seventy-seven potential BAPs were analyzed using the AnOXPP, PreAIP, and CAMPR4 databases to predict their antioxidant, anti-inflammatory, and antibacterial activities, respectively. It was estimated that five peptides, whose sequences were FAPMSRF, WMGGRL, GARCNMPKL, FIPVL, and FHPKRPWI, could exert the three bioactivities analyzed. These peptides were selected because they exhibited prediction scores higher than 0.5 for antioxidant and antibacterial activities, and above 0.38 for anti-inflammatory activity. Regarding the potential BAPs produced, an exploration of their sequences in the Uniprot Database revealed that FHPKRPWI could be released from the protein “Coatomer WD-associated region domain-containing protein” of *T. chuii* (strain PLY429; UniProtKB entry A0A7S1SM32).

The peptide GARCNMPKL could be released from the protein “formate C-acetyltransferase activity” of *Tetraselmis* sp. (strain GSL018; UniProtKB entry A0A061RIN7), and peptide FAPMSRF released from the protein “Glycosyl hydrolase family 13 catalytic domain-containing protein” of *T. chuii* (strain PLY429; UniProtKB entry A0A7S1SM91).

### 2.3. Molecular Docking of Potential Bioactive Peptides

The potential mechanisms of action of five selected peptides were evaluated through molecular docking targeting Kelch-like ECH-associated protein 1 (Keap-1), Myeloperoxidase (MPO), C-X-C motif chemokine receptor 1 (CXCR1), C-X-C motif chemokine receptor 2 (CXCR2), cytotoxin-associated gene (CagA) and vacuolating cytotoxin (VacA) proteins, in relation to their antioxidant, anti-inflammatory, and antibacterial activities against *H. pylori* infection.

Keap1 serves as a critical regulator of cellular oxidative stress response and redox homeostasis through its interaction with nuclear factor erythroid 2-related factor 2 (Nrf2). This Keap1-Nrf2 signaling pathway maintains cellular antioxidant defenses by controlling Nrf2 stability and activation [47]. Myeloperoxidase (MPO) is an enzyme in neutrophils and other phagocytic cells that uses hydrogen peroxide to produce ROS and reactive chlorine species for pathogen destruction. While essential for immune defense, excessive MPO activity can cause oxidative stress and damage to cells, contributing to inflammatory diseases. Antioxidant peptides are being developed to inhibit MPO or its reactive products to reduce such damage and control inflammation [48,49]. CXCR1 and CXCR2 are CXC chemokine receptors that mediate immune and inflammatory responses by binding to ligands like IL-8. CXCR1, mainly involved in neutrophil chemotaxis and activation, plays a key role in responses to infections such as that caused by *H. pylori*, activating pathways like the mitogen-activated protein kinase (MAPK) [50,51]. CXCR2, primarily on neutrophils and endothelial cells, also binds IL-8 and triggers intracellular signaling that promotes neutrophil migration, activation, and degranulation. Beyond host defense, CXCR2 is involved in chronic inflammation, cancer, and lung diseases due to its role in leukocyte recruitment and microenvironment modulation [52]. The CagA protein is one of the most important factors in the virulence of *H. pylori*. It is injected into host cells via the type IV secretion system (T4SS), where CagA is phosphorylated at tyrosine residues by host kinases such as Src and Csk. This phosphorylation of CagA triggers an intracellular signaling cascade that affects the cytoskeleton, which can cause alterations in cell morphology, uncontrolled proliferation, and an inflammatory response [53]. These interactions contribute to diseases such as chronic gastritis, gastric ulcers, and gastric cancer [54]. VacA is a major virulence factor secreted by *H. pylori* that facilitates persistent colonization of the gastric mucosa. It induces vacuole formation in epithelial cells, disrupts mitochondrial function, and modulates immune responses. VacA forms anion-selective channels in endosomal membranes, disrupting endosomal trafficking and cellular homeostasis, leading to cell death. Additionally, it impairs T cell activation and proliferation by interfering with key signaling pathways, aiding immune evasion [55,56].

The Gibbs free energy (ΔG) quantifies the thermodynamic feasibility of ligand-target binding interactions. As a key thermodynamic parameter, ΔG directly correlates with binding affinity. The most stable and biologically relevant interactions are typically those exhibiting the lowest ΔG values [57]. The Gibbs free energy ranges measured in our study are shown in Table 2. They were from −4.6 to −8.1 kcal/mol for Keap-1, from −6.5 to −8.0 kcal/mol for MPO, from −2.3 to −7.7 kcal/mol for CXCR1, from −5.7 to −6.5 kcal/mol for CXCR2, from −1.1 to −2.0 kcal/mol for VacA and from −2.2 to −5.3 kcal/mol for CagA, suggesting that five selected peptides could exert their possible antioxidant, anti-inflammatory and *H. pylori* pathogenicity-reducing activities through interaction with these proteins.

**Figure 4:**
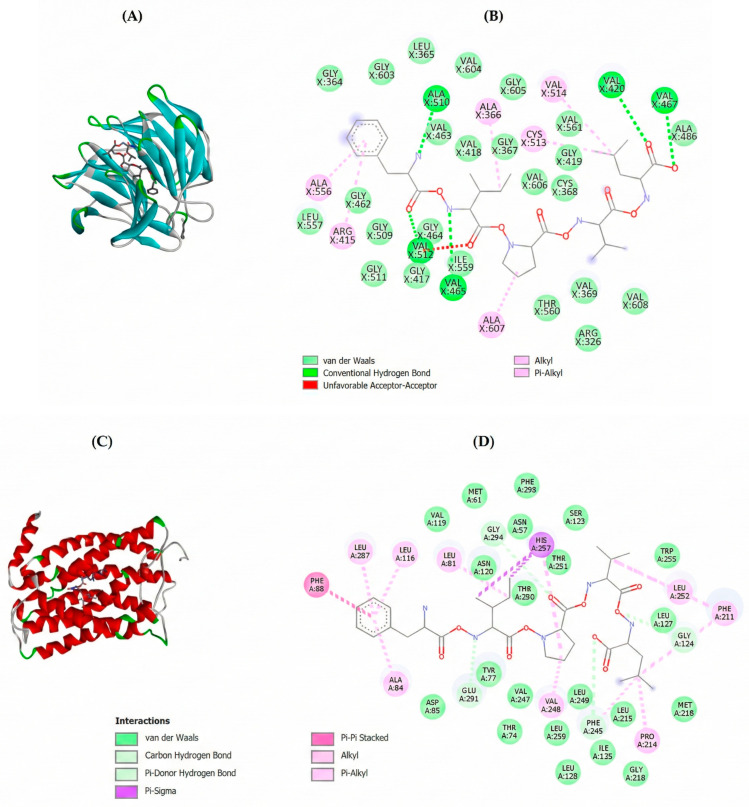

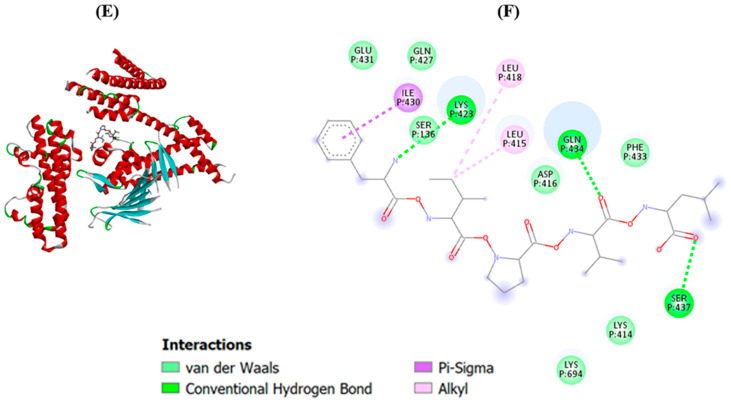
Molecular docking of the potential bioactive peptide FIPVL from *Tetraselmis chuii* digest with the human Keap-1, the CXCR1 and the CagA: (**A**) 3D molecular docking representation of FIPVL with Keap-1; Blue: beta-strands; Green: loops connecting alpha-helices and beta-strands; Gray: protein backbone or coil regions that do not form distinct secondary structural elements; (**B**) interaction type between FIPVL and Keap-1; Bright green dotted lines: conventional hydrogen bonds; Pink dotted lines: alkyl and Pi-alkyl bonds; (**C**) 3D molecular docking representation of FIPVL with CXCR1; Red: alpha-helices; Green: loops connecting alpha-helices and beta-strands; Gray: protein backbone or coil regions that do not form distinct secondary structural elements (**D**) interaction type between FIPVL and CXCR1; Bright green dotted lines: van der Waals forcesCX; Pink dotted lines: alkyl and Pi-alkyl bonds; dark pink dotted lines: Pi-Pi stacked bonds; Purple: Pi-sigma bonds; (**E**) 3D molecular docking representation of FIPVL with CagA; Blue: beta-strands; Green: loops connecting alpha-helices and beta-strands; Gray: protein backbone or coil regions that do not form distinct secondary structural elements Red: alpha-helices; (**F**) interaction type between FIPVL and CagA. Bright green dotted lines: conventional hydrogen bonds; Pink dotted lines: alkyl bonds; Purple: Pi-sigma bonds.

The molecular interactions of the peptide FIPVL with the enzymes Keap-1, CXCR1 and CagA are shown in Figure 4. In the case of Keap-1 (Figure 4A,B), the peptide showed a high binding affinity, with a Gibbs free energy of −8.1 kcal/mol. Molecular docking analysis revealed that peptide–enzyme interactions occurred through hydrogen bonds (A_510_, V_420_, V_467_, V_512_, V_465_), alkyl and Pi-alkyl interactions (V_514_, A_366_, A_556_, A_607_, C_51_3, R_415_) and van der Waals forces (Figure 4B). Under physiological conditions, Keap1 plays a critical role as a negative regulator of Nrf2 by promoting its ubiquitination and subsequent proteasomal degradation. However, the binding of specific peptides or high-affinity molecules to Keap1’s recognition site can disrupt this regulatory interaction. As a result, Nrf2 evades degradation, becomes stabilized in the cytoplasm, and can translocate into the nucleus. Once in the nucleus, Nrf2 binds to antioxidant response elements (AREs) in DNA, initiating the transcription of genes involved in oxidative stress defense, cellular detoxification, and redox homeostasis maintenance [58]. Relative to CXCR1 (Figure 4C,D), peptide FIPVL also showed a high binding affinity with a Gibbs free energy of −7.7 kcal/mol. As shown in Figure 4D, peptide–enzyme interactions occurred through carbon–hydrogen bonds and Pi-Donor hydrogen bond (G_294_, L_127_, E_291_, F_245_), Pi-sigma interactions (H_297_), Pi-Pi Stacked (F_88_), alkyl and Pi-alkyl interactions (L_287_, L_116_, L_81_, L_252_, F_211_, P_214_, A_84_, V_248_), and van der Walls forces. This receptor, a member of the G protein-coupled receptor (GPCR) family, is physiologically activated by the chemokine IL-8 or CXCL8, which plays a critical role in neutrophil chemotaxis and activation, as well as in angiogenesis and tumor progression [59].

CXCR1 activation relies on the interaction of IL-8 with its characteristic N-terminal ELR motif, which is essential to induce conformational changes in the receptor and enable the activation of intracellular signaling pathways, including calcium mobilization and ERK phosphorylation [60]. In contrast, the FIPVL peptide lacks the ELR motif and does not contain charged residues critical for receptor activation. Its markedly hydrophobic nature suggests that it acts as a competitive antagonist by occupying part or all of the extracellular binding pocket of CXCR1. Thus, FIPVL blocks IL-8 binding without inducing receptor activation, thereby preventing the recruitment and activation of inflammatory cells [61].

A high binding affinity of peptide FIPVL with CagA was also found, with a Gibbs free energy of −5.1 kcal/mol (Figure 4E,F). The peptide–enzyme interactions occurred through conventional hydrogen bonds (K_423_, Q_434_, S_437_), Pi-sigma interactions (I_430_), alkyl bonds (L_418_, L_415_) and van der Waals forces. A peptide exhibiting high binding affinity for CagA suggests stable and strong molecular interactions that may competitively inhibit the protein’s native functions. Specifically, such binding could disrupt CagA’s biological activity by targeting its EPIYA motif—the critical phosphorylation site required for host kinase interactions and subsequent pathogenic signaling [62]. This strong interaction can be beneficial for inhibiting CagA’s biological functions, blocking its ability to induce the signaling pathways responsible for the pathological effects of *H. pylori* infection. Interfering with this interaction could reduce inflammation activation, cytoskeletal disruption, and cell proliferation, all of which are crucial effects for the pathogenesis of *H. pylori* [48]. Moreover, this peptide exhibits high binding affinity for myeloperoxidase (MPO) (ΔG = −7.9 kcal/mol), forming a stable complex that may inhibit its catalytic activity and reduce HOCl formation and oxidative tissue damage [63]. It also shows high affinity for CXCR2 (ΔG = −6.4 kcal/mol), potentially blocking chemokine binding and downstream signaling, thereby inhibiting neutrophil migration and inflammatory responses [64]. Finally, the peptide binds to VacA (ΔG = −2.0 kcal/mol), interfering with host–cell binding and oligomerization, which may reduce VacA-induced vacuolation, mitochondrial dysfunction, and T-cell inhibition [65].

The peptides FAPMSRF, FHPKRPWI, GARCNMPKL, and WMGGRL consistently demonstrated higher predicted affinities for host proteins involved in inflammatory and immune responses, including MPO, CXCR1, and CXCR2. Notably, these peptides also exhibited potential interactions with VacA and CagA, suggesting a capacity to indirectly modulate the activity of these toxins while influencing host immune pathways. This selective binding profile indicates a significant potential to regulate enzymatic activity and receptor-mediated signaling in the host, impacting processes such as neutrophil recruitment, oxidative responses, and other inflammatory mechanisms. Overall, these findings underscore the potential of these peptides as modulators of host immune functions, with a particular emphasis on key proteins involved in the regulation of inflammation and bacterial virulence factors.

### 2.4. Effect of In Vitro Orogastric Digestion on Bioactivity of T. chuii

#### 2.4.1. Antioxidant Capacity Through Biochemical Assays and a Gastric Cell Model

**Figure 5:**
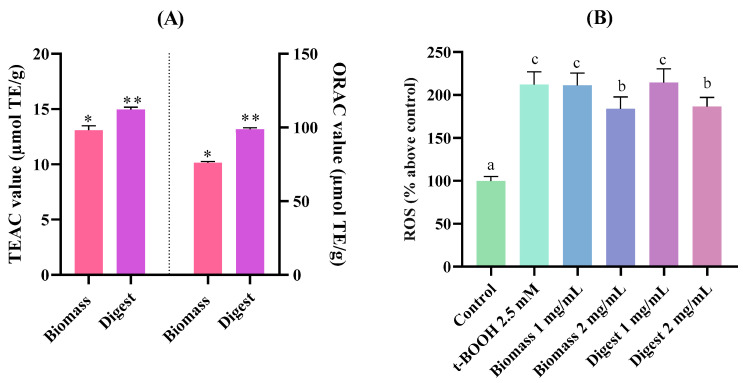
Antioxidant activity of *Tetraselmis chuii* biomass and its digest obtained after orogastric digestion through: (**A**) ABTS and ORAC (mean of the three digests obtained). TE: Trolox equivalents; (**B**) ROS levels in AGS cells stimulated with 2.5 mM t-BOOH. Different letters indicate significant differences between treatments (*p* < 0.05). Significant differences compared to control: * *p* < 0.05; ** *p* < 0.01.

An in vitro gastrointestinal digestion was carried out to evaluate the impact of digestive enzymes on the bioactivity of *T. chuii* biomass. The results of antioxidant activity using the ORAC and ABTS methods are shown in Figure 5A,B. The whole biomass presented a TEAC value of 13.10 ± 0.25 μmol TE/g sample and an ORAC value of 76.01 ± 0.98 μmol TE/g sample, suggesting the presence of diverse antioxidant compounds in the biomass. Following orogastric digestion, the TEAC and ORAC values increased significantly (*p* < 0.05) to 14.96 ± 0.21 and 98.83 ± 0.37 μmol TE/g sample, respectively. The increased radical-scavenging capacity detected by both biochemical methods suggested that BAPs were released after the action of saliva and gastric enzymes, contributing to the antioxidant activity shown by endogenous antioxidant compounds present in the biomass. Our in silico analysis confirmed the release of potential antioxidant peptides from microalgae proteins by the action of pepsin.

The protective effects of the *T. chuii* biomass and its orogastric digest against oxidative stress and inflammation induced by *H. pylori* were also assessed using a human gastric AGS cell model. First, concentrations of the extract (2 and 1 mg/mL) were tested to evaluate whether any of them affected the viability of AGS cells. Both doses maintained cell viability above 80%, thus, they were selected for the following assays. Previous studies on microalgae cytotoxicity across different cell lines have revealed significant differences between species. These variations likely stem from each microalgae’s specific biochemical composition, the type of extract used, and the inherent sensitivity of the cell lines employed in the assays. Thus, Sansone et al. [66] investigated the effects of an ethanol–water extract from *T. suecica* on lung adenocarcinoma cells’ viability, observing a slight cytotoxic effect at 0.4 mg/mL.

In order to study the protective effect of *T. chuii* and its digest against oxidative stress, the ROS levels were measured in chemical-stimulated AGS cells (Figure 5B). At the highest assayed dose of 2.0 mg/mL, both biomass and digest reverted significantly (*p* < 0.05) the ROS-inducing effect exerted by t-BOOH by 1.83 and 1.86 times, respectively. The extract of *T. chuii* exhibits a high antioxidant potential, which contributes to the inhibition of ROS [67]. Recent research highlights the antioxidant potential of BAPs derived from microalgae, such as *C. vulgaris* and *A. platensis*, as an effective natural defense mechanism against ROS, thanks to their richness in AAs like cysteine (C), histidine (H), and glutamic acid (E), which play critical roles in scavenging and cellular protection [68]. In addition, this microalga produces superoxide dismutase (SOD), a crucial enzyme involved in the elimination of ROS and the protection against oxidative stress [69]. In a study carried out by Galasso et al. [70], the ROS production in a prostate tissue cell line (PNT2) treated with *T. suecica* extracts and 2,2-diphenyl-1-picrylhydrazyl (DPPH) was evaluated. The extracts significantly reduced ROS levels, intracellular oxidative stress, and apoptosis at all assayed doses.

#### 2.4.2. Protective Antioxidant and Anti-Inflammatory Effects in *H. pylori*-Infected AGS Cells

**Figure 6:**
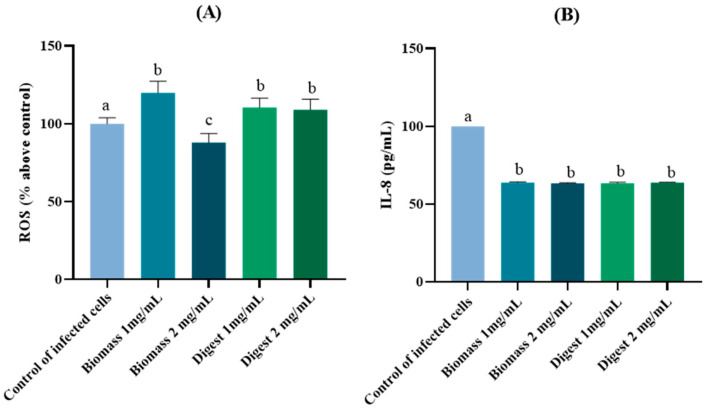
Effect of *Tetraselmis chuii* biomass and digest on: (**A**) reactive oxygen species (ROS) levels and (**B**) interleukin (IL)-8 production in *H. pylori*-infected AGS cells. Different letters indicate significant differences between treatments (*p* < 0.05).

The effect of the *T. chuii* biomass and its orogastric digest on ROS levels in *H. pylori*-infected AGS cells is shown in Figure 6A.

Treatment of *H. pylori*-infected cells with 1 mg/mL of biomass led to an increase in ROS production, whereas the same biomass at 2 mg/mL significantly (*p* < 0.05) reduced ROS production compared to the infected control (12% of reduction). However, digested biomass, regardless of its concentration, did not cause a reduction in ROS production. These results suggest that the biomass contains both pro-oxidant and antioxidant compounds, with the latter only exhibiting antioxidant activity at the higher concentration. This reduction in ROS production was affected by orogastric digestion, indicating that the antioxidant compounds involved—regardless of their nature—were sensitive to this process. In the case of a likely peptide fraction with antioxidant activity, the antioxidant potential of peptides can be significantly affected by orogastric digestion. This is because the digestive environment, including enzymatic activity and pH changes, could alter the structure and stability of peptides, potentially impairing their antioxidant function [71]. Further studies should be needed to identify these compounds and assess whether their protection through encapsulation techniques is feasible.

Figure 6B illustrates the effect of *T. chuii* biomass and its digest on IL-8 production. This cytokine is the main pro-inflammatory marker associated with *H. pylori* infection by gastric cells. In all cases, production of IL-8 significantly (*p* < 0.05) decreased, regardless of the biomass concentration or whether it had undergone orogastric digestion. The resistance of the anti-inflammatory compounds to digestion suggested that they differ from those involved in ROS modulation. The compounds responsible for the anti-inflammatory activity of *T. chuii* biomass might include several bioactive molecules known to be resistant to digestive degradation. Although the peptide fraction in this study appears to lose its antioxidant activity post-digestion, it is possible that distinct peptide fractions retain anti-inflammatory properties [72]. SOD, which has been identified in *T. chuii,* has been reported to modulate inflammatory responses by regulating the expression of both pro- and anti-inflammatory cytokines [67]. Carotenoids such as lutein and β-carotene are generally stable during digestion and might exert modulatory effects on inflammation [73]. Additionally, *T. chuii* contains omega-3, omega-6, and omega-9 polyunsaturated fatty acids (PUFA), which are well known for their anti-inflammatory effects and relative resistance to digestive processes [74]. Finally, although less extensively studied in *T. chuii*, certain phenolic compounds might also be present and contribute to its anti-inflammatory activity [75].

#### 2.4.3. Antibacterial Activity

Some BAPs derived from microalgae have demonstrated activity against Gram-negative bacteria [76]. Although no studies have specifically evaluated the antibacterial activity of *T. chuii* against *H. pylori*. Guzmán et al. [11] reported the presence of bactericidal peptides against both Gram-positive and Gram-negative pathogenic bacteria in the microalgae *T. suecica*. with bactericidal effects against both Gram-positive and Gram-negative pathogenic bacteria. These findings suggest a possible antibacterial effect against *H. pylori*. However, in the present study, no significant (*p* > 0.05) antibacterial activity was observed for either *T. chuii* biomass (5.47 ± 0.07 log CFU/mL) or its orogastric digest (5.34 ± 0.13 log CFU/mL) in comparison with the growth control (6.11 ± 0.01 log CFU/mL). A plausible explanation for the reduced antibacterial activity observed in the in vitro experiments is the insufficient peptide concentration used during the assays, which may have been below the minimum inhibitory threshold despite molecular docking results indicating strong binding affinity to the predicted biological target. Low peptide concentrations can markedly diminish antibacterial efficacy because they may fail to disrupt bacterial membranes—a key mechanism of action for many BAPs [77]. Another contributing factor could be enzymatic degradation of the peptides by proteases present in the culture medium or introduced during sample handling and preparation. Such degradation likely reduces the effective peptide concentration, thereby limiting its ability to interact with the target microorganism [78]. This discrepancy highlights the limitations of relying solely on computational predictions, which do not account for critical physicochemical and biological factors such as peptide stability, solubility, bioavailability, and the complexity of the experimental environment. Therefore, while docking studies provide valuable preliminary insights, they must be complemented by rigorous experimental validation to accurately assess the antibacterial potential of peptide-based compounds. Other compounds with potential antibacterial activity, such as phenolic compounds [75] and PUFAs [79], that could also be present in the microalgae, did not show any effect against *H. pylori*, likely due to their concentration in the sample.

## 3. Materials and Methods

### 3.1. Microalgae Biomass and Its Pre-Treatment

SOD-rich *T. chuii* biomass (TetraSOD^®^) was provided by Fitoplancton Marino, S.L. (El Puerto de Santa María, Cadiz, Spain). It was subjected to a combined sequential pre-treatment consisting of cycles of freezing (−20 °C, 15 h) and thawing (room temperature, 9 h) for five consecutive days, followed by ultrasonic (US) treatment. The US treatment was conducted with the Fisherbrand™ Sonicador 505 (Danbury, CT, USA) operating at 70% amplitude (20 kHz, 500 W) using a 0.63 cm probe. The process consisted of six cycles, each lasting 20 s, with a 1 min resting period between cycles.

### 3.2. Protein Characterization of T. chuii

Protein content was measured using the bicinchoninic acid (BCA) method, following the instructions of the manufacturer of the Thermo Scientific™ Pierce™ BCA kit (Waltham, MA, USA). Bovine serum albumin (BSA) at concentrations between 25 and 1000 μg/mL was used as a standard. The absorbance was measured at 562 nm on the plate reader Biotek Synergy HT (Winooski, VT, USA). All samples were analyzed in triplicate, and the results were expressed as a percentage of total protein (%).

The protein profile was determined by gel electrophoresis (SDS-PAGE), following the protocol described by Paterson et al. [23]. The samples were diluted to 1.25 mg of protein/mL in a specific buffer and subjected to heat treatment (100 °C for 5 min) and centrifugation at 1100 rpm for 10 s. A Criterion^TM^ Cell system (Bio-Rad, Hercules, CA, USA) was used for running gels. 25 µL of the sample (50 µg of protein) was loaded onto a 12% Bis-Tris Criterion™ XT Precast Gel polyacrylamide gel (Bio-Rad). The electrophoretic migration was carried out at 100 V for 5 min and at 150 V for 1 h. After its staining, the gel image was captured using the Molecular Imager^®^ Versadoc™ MP 4000 and the image was analyzed using Image Lab 6.1 software (Bio-Rad).

### 3.3. Proteomic and Functional Analysis

A suspension of the biomass of *T. chuii* in 50 μL of sample buffer was prepared and loaded into 1.2 cm wide wells of a standard SDS-PAGE gel (0.75 mm thick, 4% stacking, and 10% resolving; Bio-Rad), following a previously described procedure [80]. The electrophoresis was halted when the leading edge migrated 3 mm into the resolving gel, concentrating the entire proteome at the stacking/resolving interface. The unseparated protein bands were stained with Coomassie (Bio-Rad), excised, cut into 2 × 2 mm cubes, and transferred into 0.5 mL microcentrifuge tubes [81].

The gel pieces were destained using a mixture of acetonitrile (ACN) and H_2_O (1:1, *v*:*v*), then reduced and alkylated. Disulfide bonds were reduced with 10 mM 1,4-dithiothreitol (DTT) at 56 °C for 1 h, and thiol groups were alkylated with 10 mM iodoacetamide for 30 min in the dark at room temperature. Protein digestion was performed in situ with sequencing-grade trypsin (Promega, Madison, WI, USA) as per Shevchenko et al.’s protocol [82]. The gel pieces were dehydrated by removing the liquid with ACN, dried in a speedvac, and rehydrated in 100 mM Tris-HCl (pH 8) containing 10 mM CaCl_2_ and 60 ng/mL trypsin at a 5:1 protein-to-enzyme (*w*/*w*) ratio. The samples were incubated on ice for 2 h, then at 37 °C for 12 h. Digestion was terminated with the addition of 1% trifluoroacetic acid (TFA). The supernatants were dried and desalted using OMIX Pipette tips C18 (Agilent Technologies, Santa Clara, CA, USA) before analysis. The digestion was performed in the presence of 0.2% RapiGest (Waters, Milford, MA, USA).

The desalted digest was dried, resuspended in 10 μL of 0.1% formic acid, and analyzed by RP-LC-MS/MS using an Easy-nLC 1200 system coupled to an ion trap LTQ-Orbitrap-Velos-Pro hybrid mass spectrometer (Thermo Scientific, Waltham, MA, USA). Peptides were concentrated on-line using a 0.1 mm × 20 mm C18 RP precolumn (Thermo Scientific) and separated on a 0.075 mm × 250 mm bioZen 2.6 μm Peptide XB-C18 RP column (Phenomenex, Torrance, CA, USA) running at 0.25 μL/min. The elution was performed with a 180 min dual gradient: 5–25% solvent B for 135 min, 25–40% solvent B for 45 min, 40–100% solvent B for 2 min, and 100% solvent B for 18 min (Solvent A: 0.1% formic acid in water; Solvent B: 0.1% formic acid, 80% ACN in water).

Electrospray ionization (ESI) was conducted using a Nano-bore Stainless Steel emitter with a 30 μm inner diameter (Proxeon, Odense, Denmark) at a spray voltage of 2.1 kV and an S-Lens setting of 60%. Orbitrap resolution was set at 30,000 [83]. Peptides were detected in survey scans from 400 to 1600 amu (1 μscan), followed by twenty data-dependent MS/MS scans (Top 20), using an isolation width of 2 u, normalized collision energy of 35%, and dynamic exclusion for 60 s. Charge-state screening was applied to reject unassigned and singly charged ions.

Peptide identification from the raw data was performed using the PEAKS Studio v11.5 search engine (Bioinformatics Solutions Inc., Waterloo, ON, Canada). The database search was conducted against the UniProt-*Tetraselmis chuii*. fasta (13,477 entries; UniProt release 11/2023). The following search parameters were applied: tryptic cleavage (semi-specific) after arginine (R) and lysine (K), allowing up to two missed cleavages, with tolerances of 20 ppm for precursor ions and 0.6 Da for MS/MS fragment ions. Methionine (M) oxidation and cysteine (C) carbamidomethylation were considered as optional modifications. The false discovery rates (FDRs) for PSMs and proteins were limited to 0.01. Only proteins identified by at least two unique peptides from LC/MS/MS analyses were considered confidently identified [84,85,86].

The functional analysis of the identified proteins from *T. chuii* biomass was performed using the functional analysis module of Omicsbox 3.3.2 software (Biobam, Valencia, Spain), following the steps detailed in Supplementary Figure S1. Proteins were classified into cellular components, molecular function, and biological process groups through the BLAST2GO methodology. The database for alignment was non-redundant protein sequences (nr v5), filtering by taxonomy: *T. chuii* (code: 63592) [87].

### 3.4. In Silico Orogastric Digestion of Microalgae Proteins: Potential Bioactive Effects

The identified proteins were subjected to an in silico gastric digestion using the Rapid Peptides Generator (RPG) software (Pasteur Institute, Paris, France) v 22.2.3 [88]. Using the Python system (3.12) and data in FASTA format, digestion was simulated using pepsin as the gastric enzyme (code 34). Following the removal of free AAs and duplicate peptides, the remaining peptides were classified by (i) the number of AAs, (ii) molecular weight (MW), and (iii) type of AA. Moreover, peptides were processed using the server based on a novel N-to-1 neural network Peptide Ranker [18] to classify them according to their bioactivity. Peptides with a probability of being bioactive ≥ 0.8 value were selected to estimate their (i) antioxidant activity by using the AnOxPP software [89]; (ii) antimicrobial activity with CAMPR4 software [90], and (iii) anti-inflammatory activity using PreAIP software [91].

### 3.5. Molecular Docking

Molecular interactions were predicted through molecular docking for the selected peptide sequences and the catalytic sites of Keap-1, MPO, CXCR1, CXCR2, VacA hexamer and CagA. The crystal structures for Keap-1 (2FLU), MPO (3F9P), CXCR1 (2LNL), CXCR2 (4N6X), VacA (6ODY) and CagA (4DVY) were retrieved from the Protein Data Bank (http://www.rcsb.org/ accessed on 25 November 2025) [92]. Peptides were designed using Marvin Sketch software (Chemaxon, version 19.22.0). Flexible torsions, charge assignments, and grid sizes were configured using AutoDock Tools v1.1.2. Molecular docking was performed using AutoDock Vina v1.1.2 [93]. The binding pose with the lowest binding energy was selected as the representative conformation and visualized using the Discovery Studio 2016 Client (Dassault Systemes Biovia Corp. R, Velizy-Villacoublay, France). The binding pose with the lowest binding energy was selected as the representative conformation and visualized using the Discovery Studio 2016 Client.

### 3.6. In Vitro Simulated Orogastric Digestion

The orogastric digestion simulation of the pre-treated biomass of *T. chuii* was performed following the international INFOGEST protocol with some modifications [94]. 1 g of microalgae biomass was dissolved in 500 µL of deionized water and 4.5 mL of human salivary fluid and incubated at 37 °C for 2 min. For the gastric phase, 4.8 mL of simulated gastric fluid (SGF), 3 µL of CaCl_2_, and 300 µL of rabbit gastric extract (RGE15, lipase: ≥15 U/mg and pepsin: ≥500 U/mg, Sigma-Aldrich, St. Louis, MO, USA) were added. The pH was adjusted to 3.0, and the mixture was incubated at 37 °C for 2 h. To terminate the digestion, the pH was adjusted to 7.0–7.5 and heated at 95 °C for 5 min. The digests were freeze-dried and stored at −20 °C.

### 3.7. Biological Properties of T. chuii Biomass and Its Orogastric Digests

#### 3.7.1. Antioxidant Activity (ABTS and ORAC Assays)

The antioxidant activity of the samples was evaluated using the 2,2′-azino-bis-(3-ethylbenzothiazolin-6-ammonium sulfonate) (ABTS, Sigma-Aldrich) radical neutralization assay [95] and the oxygen radical absorbance capacity (ORAC) assay [96]. The ABTS + radical was generated by combining ABTS with potassium persulfate (K_2_S_2_O_8_) and incubating overnight in the dark. 6-hydroxy-2,5,7,8-tetramethylchroman-2-carboxylic acid (Trolox, Sigma-Aldrich) served as a standard at concentrations between 25 and 200 μM. Samples were prepared in 5 mM phosphate-buffered saline (PBS, pH 7.4, Corning-Fisher Scientific, Waltham, MA, USA) at concentrations ranging from 2 to 15 mg/mL (biomass) and 2 to 10 mg/mL (digests). Each sample (20 μL) was added to a 96-well plate with 180 μL of ABTS + solution (adjusted to an absorbance of 0.70 ± 0.02), incubated for 5 min in the dark, and the absorbance was measured at 734 nm on the plate reader Biotek Synergy HT.

For the ORAC assay, Trolox was used as a standard at concentrations between 1 and 8 µM. Samples were prepared in 0.075 M PBS buffer at concentrations ranging from 1.25 mg/mL to 0.156 mg/mL. Each sample (20 μL) was added to a 96-well black plate with 120 μL of fluorescein disodium (FL, Sigma-Aldrich) solution (116.61 nM), incubated for 10 min at 37 °C, followed by the addition of 60 μL of 2′-azobis (2-amidinopropane) dichlorohydrochloride (AAPH, Sigma-Aldrich) solution (0.048 mM). Control wells received PBS instead of AAPH. The plate was incubated for 95 min at 37 °C, measuring the fluorescence at 485 nm (λ_exc_) and 520 nm (λ_em_) at the Fluostar Optima BMG Labtech (Ortenberg, Germany) plate reader. In both ABTS and ORAC assays, the results were expressed as μmol Trolox equivalents (TE)/g of sample and analyzed in triplicate (*n* = 3).

#### 3.7.2. Antibacterial Activity Against *H. pylori*

*H. pylori* 19449 was sourced from the American Type Culture Collection (ATCC) in Manassas, VA, USA. The strain was preserved at −80 °C in Brucella Broth (BB) (Becton, Dickinson & Co., Madrid, Spain) supplemented with 20% glycerol. For agar plating, Müeller-Hinton agar enriched with 5% defibrinated sheep blood (MHB) (Becton, Dickin-son & Co.) was employed. Liquid cultures were grown in BB supplemented with 10% horse serum (HS) (Biowest, Barcelona, Spain). The inoculum preparation for the *H. pylori* strain followed the protocol described by Silvan et al. [97]. Frozen strain was reactivated by inoculation (200 µL) in an MHB plate and incubation in a Variable Atmosphere Incubator (VAIN) (85% N_2_, 10% CO_2_ and 5% O_2_) (MACS-VA500, Don Whitley Scientific, Bingley, UK) at 37 °C for 72 h. Bacterial grown from one MHB plate were suspended in 2 mL of BB + 10% HS or culture medium cell (~1 × 10^8^ colony forming units, CFU/mL) and used as bacterial inoculum in the different assays.

The antibacterial capacity of the samples against *H. pylori* was performed by a quantitative method following the protocol described by Silvan et al. [97]. 1 mL of the samples (final concentration of 20 mg/mL), dissolved in BB supplemented with 10% HS, was filtered using a 0.22 μm filter (B. Braun, Melsungen, Germany). Filtrate was diluted in 3.9 mL of BB with 100 μL of bacterial inoculum (~1.5 × 10^6^ CFU/mL) and incubated for 24 h at 37 °C and 150 rpm in a microaerophilic atmosphere using a variable atmosphere incubator (VAIN) (85% N_2_, 10% CO_2_, 5% O_2_) (MACS-VA500, Don Whitley Scientific, Bingley, UK). Positive and negative growth controls were prepared by transferring 4.9 mL of BB supplemented with 10% HS and 100 μL of bacterial inoculum, and 5 mL of BB, respectively. After incubation, serial dilutions of the mixtures were prepared with a 0.9% NaCl saline solution and seeded at a volume of 20 μL on Müeller-Hinton agar plates supplemented with 5% defibrinated sheep’s blood and incubated in a microaerophilic atmosphere at 37 °C for 72 h. All samples were analyzed in quadruplicate (n = 4). Finally, the number of CFU was measured, and the results were expressed as log CFU/mL.

#### 3.7.3. Antioxidant and Anti-Inflammatory Effects in Human Gastric Cells

##### Human Gastric Cells (AGS) Culture

Human gastric adenocarcinoma cells (AGS, ATCC CRL-1739 ™) were cultured in Dulbecco’s Modified Eagle Medium/F12 (DMEM/F12, Corning–Fisher Scientific) with L-glutamine, HEPES (Corning-Fisher Scientific), 10% fetal bovine serum (FBS, Lonza Group Ltd., Basel, Switzerland), and 1% penicillin/streptomycin/amphotericin B (PSA) solution (Biowest). They were maintained at 37 °C with 5% CO_2_ and 95% air under constant humidity.

##### Cell Viability

The evaluation of the effects of biomass and digests on cell viability was conducted using the 3-(4,5-dimethylthiazol-2-yl)-2,5-diphenyltetrazol bromide (MTT) assay, following the protocol described by Franca-Oliveira et al. [98]. AGS cells were seeded (2 × 10^5^ cells/well) in 96-well plates and incubated at 37 °C for 24 h. The culture medium was removed, and 120 μL of the samples in DMEM/F12 without FBS (at 0.125–2 mg/mL concentrations) were added and incubated for 2 h at 37 °C. After treatment, samples were removed, MTT (Sigma-Aldrich) solution (0.5 mg/mL) in culture medium was added, and cells were incubated for another 2 h. Formazan crystals formed were dissolved in dimethyl sulfoxide (DMSO), and the absorbance was read at 570 nm using the Multiskan FC plate reader. Samples were analyzed in at least 8 wells (n = 8), with results expressed as a percentage of viable cells (control set at 100%).

##### Effects on Reactive Oxygen Species (ROS) Production

The effect of the samples on the cellular ROS production was evaluated in AGS cells stimulated with tert-butyl hydroperoxide (t-BOOH, Sigma-Aldrich) or infected with *H. pylori*, and using 2′,7′-dichlorofluorescein diacetate (DCFH-DA, Sigma-Aldrich) as a fluorescent probe [99]. Cells were seeded in 24-well plates (1 × 10^5^ cells/well) and incubated for 24 h. After removing the media and washing with PBS, cells were treated for 2 h with biomass or orogastric digest dissolved in DMEM/F12 without FBS, at concentrations of 1 mg/mL and 2 mg/mL, under basal and stimulated conditions, and at concentrations between 0.5 mg/mL and 2 mg/mL for *H. pylori*-infected cells. After washing, they were incubated with DCFH-DA for 30 min and washed with PBS. Depending on the condition, cells were exposed to DMEM/F12 without FBS (basal condition), 2.5 mM t-BOOH (oxidized cells), or *H. pylori* (density of 1.50 ± 0.81 × 10^6^ CFU/mL). Cells were incubated for 3 h, measuring the fluorescence every 90 min at 485 nm (λ_exc_) and 520 nm (λ_em_), in the Fluostar Optima BMG Labtech plate reader. Samples were analyzed in at least 6 wells (n = 6), with results expressed as a percentage of ROS levels (control set at 100%).

##### Effects on Interleukin (IL)-8 Production

To determine the impact of biomass and digests on the IL-8 production in *H. pylori*-infected AGS cells, a human anti-IL-8 ELISA analysis was performed using the Diaclone SAS kit (Besancon Cedex, France). AGS cells were seeded in 96-well plates at 5 × 10^5^ cells/mL and incubated for 24 h at 37 °C. The cells were treated with biomass or its orogastric digest for 2 h, at concentrations between 0.5 mg/mL and 2 mg/mL and washed with PBS. To infect cells, *H. pylori* was dissolved in DMEM/F12 without FBS or antibiotics, at a density of 2.21 ± 0.11 × 10^9^ CFU/mL and incubated with the cells for 24 h. Supernatants were collected to measure IL-8 following the manufacturer’s instructions, using a standard diluent with concentrations ranging from 0 to 1000 pg/mL. The values of IL-8 production by AGS cells were calculated by extrapolation in the standard line, expressing the results in pg/mL. Samples were analyzed in triplicate (n = 3).

### 3.8. Statistical Analysis

Statistical analyses were conducted using GraphPad Prism version 8.0 (GraphPad Software, San Diego, CA, USA). Differences among groups were evaluated by one-way analysis of variance (ANOVA). When the ANOVA indicated a significant effect, pairwise comparisons between each experimental group and the control were performed using Dunnett’s post hoc test. *p*-values less than 0.05 were considered statistically significant.

## 4. Conclusions

This study presents a comprehensive proteomic characterization of *T. chuii* biomass and explores its potential as a source of BAPs with antioxidant, anti-inflammatory, and antibacterial activities against *H. pylori*. In silico digestion allowed the modeling of the release of peptide sequences with high bioactivity scores, and molecular docking confirmed stable interactions of five selected peptides with key proteins involved in oxidative stress, inflammation, and *H. pylori* virulence. Although *T. chuii* or its in vitro orogastric digest did not exert significant antibacterial activity against *H. pylori*, they showed antioxidant and anti-inflammatory effects in both biochemical and cell culture models, highlighting the potential value of compounds present in microalgae *T. chuii* to mitigate the oxidative stress and/or inflammation associated with *H. pylori* infection. Thus, our results support the use of *T. chuii* as a promising candidate for the development of functional food ingredients and/or nutraceuticals aimed at managing this infection. Future research should focus on identifying the responsible compounds of the observed effects, elucidating their mechanisms of action and enhancing their stability to confirm their bioactivity in an in vivo model.

## Figures and Tables

**Table 1:** Number of peptides generated during in silico digestion with pepsin from identified *Tetraselmis chuii* proteins, releasing more than 75 peptides/protein.

Accession ^a^	−10logP ^b^	Average Mass (kDa)	Description ^c^	Peptides Generated After in Silico Gastric Digestion
A0A7S1SXE6	362.47	99.05	Tr-type G domain-containing protein	93
A0A7S1SM83	346.30	101.61	Alpha-14 glucan phosphorylase	105
A0A7S1SNL9	339.13	84.11	Aconitate hydratase mitochondrial	76
A0A7S1SIF9	317.63	92.59	formate C-acetyltransferase	90
A0A7S1X996	313.38	91.33	ACT domain-containing protein (Fragment)	75
A0A7S1SHH8	305.65	100.13	ABC transporter domain-containing protein	89
A0A7S1X1T7	289.92	129.14	Cation-transporting P-type ATPase N-terminal domain-containing protein	107
A0A7S1SVE5	284.05	66.20	Alpha-14 glucan phosphorylase (Fragment)	82
A0A7S1SPX6	265.75	98.81	Coatomer subunit gamma	81
A0A7S1T3I0	261.36	88.82	Prolyl endopeptidase	102
A0A7S1SPT9	234.30	104.32	carbamoyl-phosphate synthase (glutamine-hydrolyzing)	96
A0A7S1X6A5	231.33	103.28	Cation-transporting P-type ATPase N-terminal domain-containing protein	92
A0A7S1SQY6	220.37	109.36	Clp R domain-containing protein	92
A0A7S1SKB1	202.03	113.48	Uncharacterized protein	100
A0A7S1T064	186.71	155.40	magnesium chelatase	146
A0A7S1SWQ6	181.90	178.52	SUEL-type lectin domain-containing protein	171
A0A7S1WYL3	171.80	105.99	leucine--tRNA ligase (Fragment)	89
A0A7S1SW31	165.90	111.31	Pyruvate phosphate dikinase AMP/ATP-binding domain-containing protein	120
A0A7S1SM91	140.91	101.38	Glycosyl hydrolase family 13 catalytic domain-containing protein (Fragment)	103
A0A7S1X1F6	139.34	95.70	valine--tRNA ligase	85
A0A7S1SM32	107.70	99.61	Coatomer WD-associated region domain-containing protein (Fragment)	89

^a^ Accession number of the protein as obtained from the FASTA database. ^b^ PEAKS protein score calculated as the weighted sum of the −10log P scores of the proteins supporting peptides. ^c^ Description of proteins as obtained from the FASTA database.

**Table 2:** Estimated free binding energy (Kcal/mol) among protein peptide sequences of *Tetraselmis chuii* and the catalytic site of diverse enzymes.

Peptide	Free Binding Energy (Kcal/mol)					
	Keap-1	MPO	CXCR1	CXCR2	VacA	CagA
FAPMSRF	−4.6	−7.5	−6.9	−6.5	−1.3	−2.2
FHPKRPWI	n.d.	−8.0	−2.3	−6.5	−1.2	n.d.
FIPVL	−8.1	−7.9	−7.7	−6.4	−2.0	−5.1
GARCNMPKL	n.d	−6.5	−4.5	−5.7	−1.1	n.d.
WMGGRL	−7.3	−7.2	−5.5	−6.3	−1.6	−5.3

Negative free energy values indicate greater affinity between the peptide and the protein. The most probable interactions are identified based on the lowest binding energy, as well as the presence of hydrogen bonds, hydrophobic interactions, and salt bridges at the active site. **Keap-1**: Kelch-like ECH-associated protein 1; **MPO**: Myeloperoxidase; **CXCR1**: C-X-C motif chemokine receptor 1; **CXCR2**: C-X-C motif chemokine receptor 2; **VacA**: vacuolating cytotoxin; **CagA**: cytotoxin-associated gene A; **n.d:** undetected.

## Data Availability

The original contributions presented in this study are included in the article/Supplementary Materials. Further inquiries can be directed to the corresponding authors.

## Supplementary Materials

The following supporting information can be downloaded at: https://www.mdpi.com/article/10.3390/molecules30244669/s1, Supplementary Figure S1: Complete Omicsbox workflow followed for the proteomic functional analysis of *Tetraselmis chuii*. Supplementary Figure S2: Complete functional distribution of detected proteins from *Tetraselmis chuii* biomass in biological process functional group using gene ontology (GO). Supplementary Figure S3: Complete functional distribution of detected proteins from *Tetraselmis chuii* biomass in molecular function functional group using gene ontology (GO). Supplementary Figure S4: Complete functional distribution of detected proteins from *Tetraselmis chuii* biomass in cellular component functional group using gene ontology (GO). Supplementary Table S1: Identified proteins from *Tetraselmis chuii*.
