# Supplementary material for: Tetraselmis chuii as Source of Bioactive Compounds Against Helicobacter pylori: An Integrated Proteomic and Bioactivity Approach
Source: Molecules. 2025 Dec 5;30(24):4669. doi: 10.3390/molecules30244669 (PMC12736364; doi:10.3390/molecules30244669)
Supplement: Supplementary file 1 [file molecules-30-04669-s001.zip › Supplementary Upload-Revision/molecules-3995090-supplementary -1/Supplementary Figures_Majchrzak.pdf]

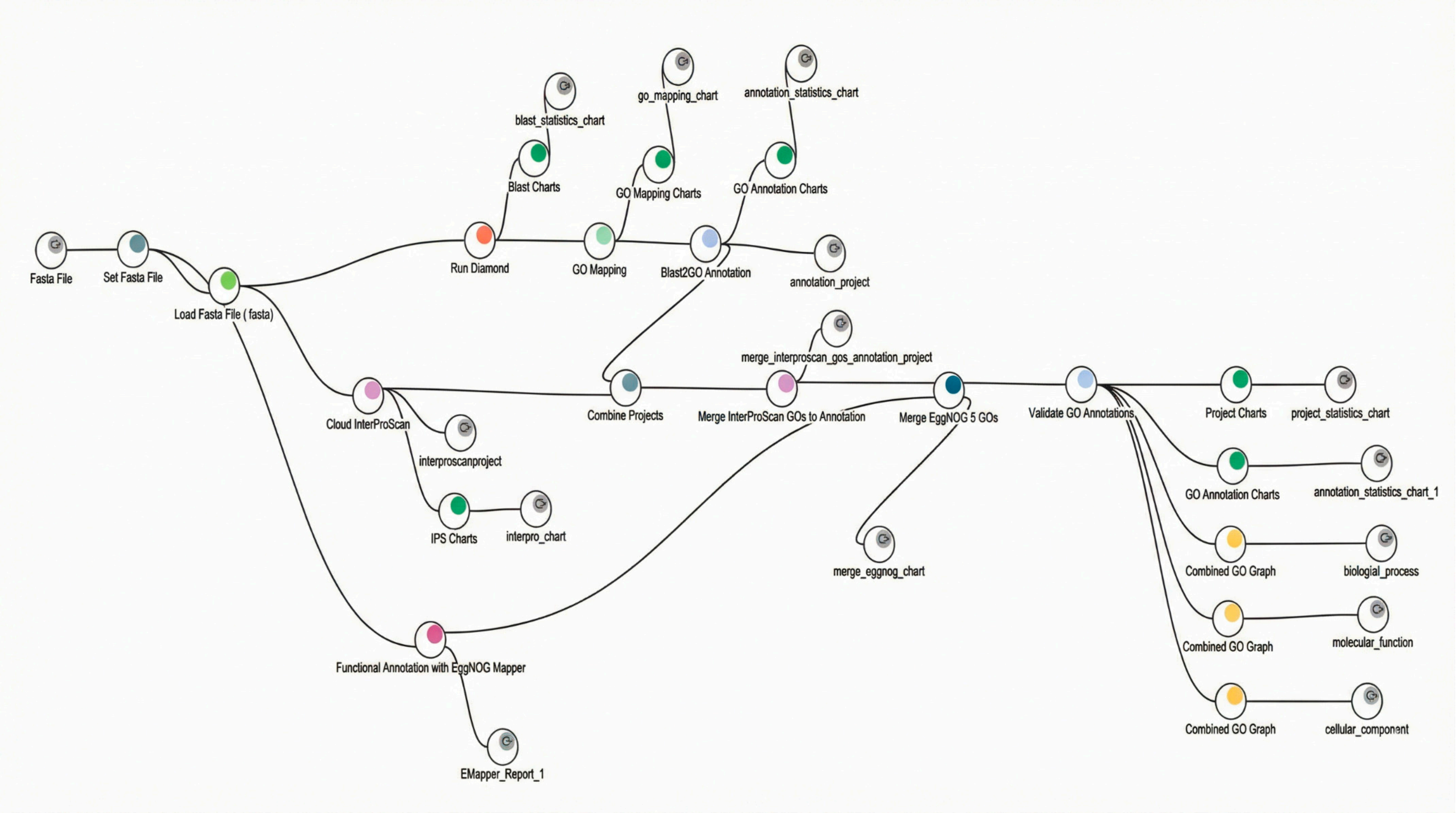

Supplementary Figure S1. Complete Omicsbox workflow followed for the proteomic functional analysis of *Tetraselmis chuii*.

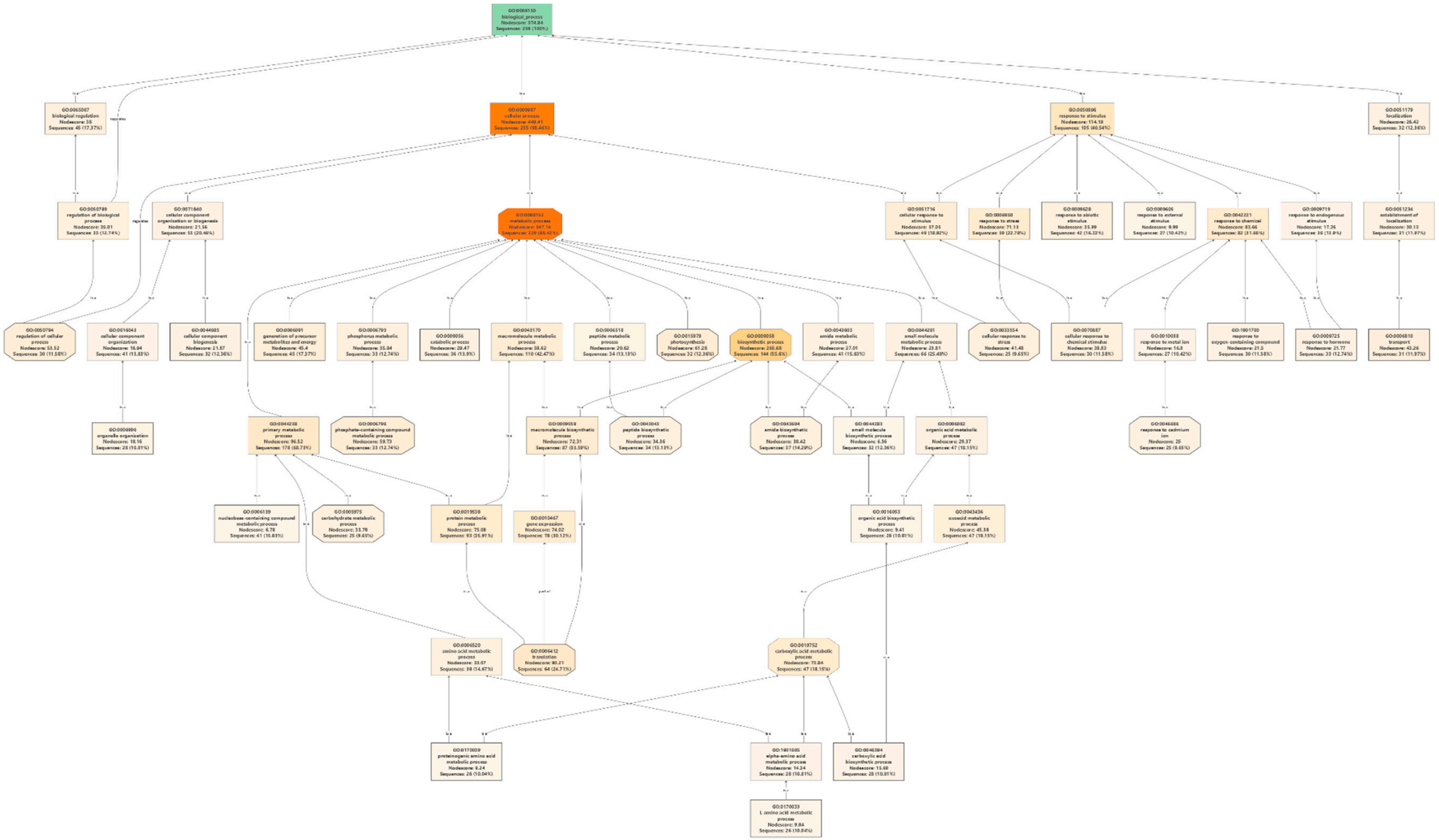

Supplementary Figure S2: Complete functional distribution of detected proteins from *Tetraselmis chuii biomass* in biological process functional group using gene ontology (GO)

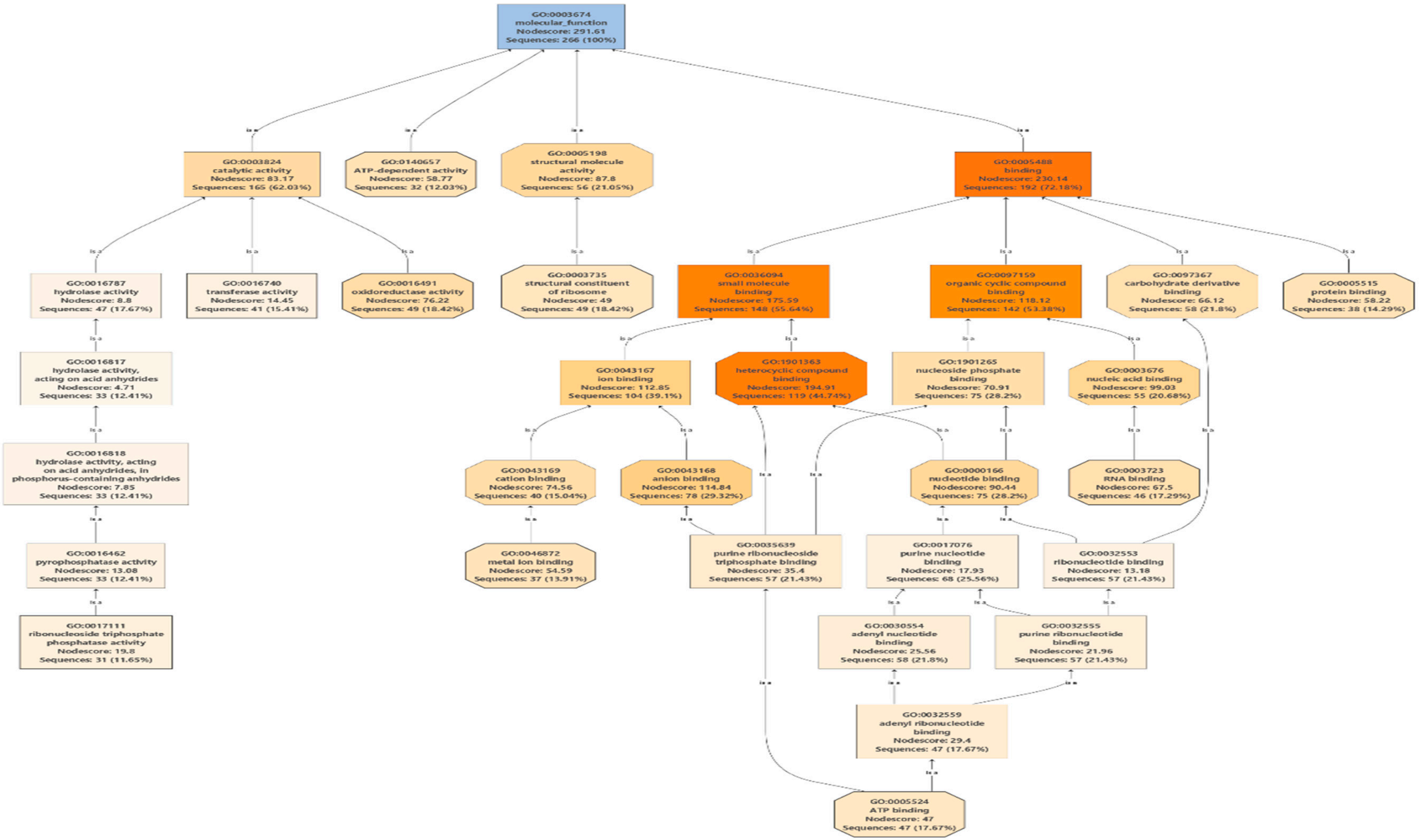

Supplementary Figure S3: Complete functional distribution of detected proteins from *Tetraselmis chuii* biomass in molecular function functional group using gene ontology (GO).

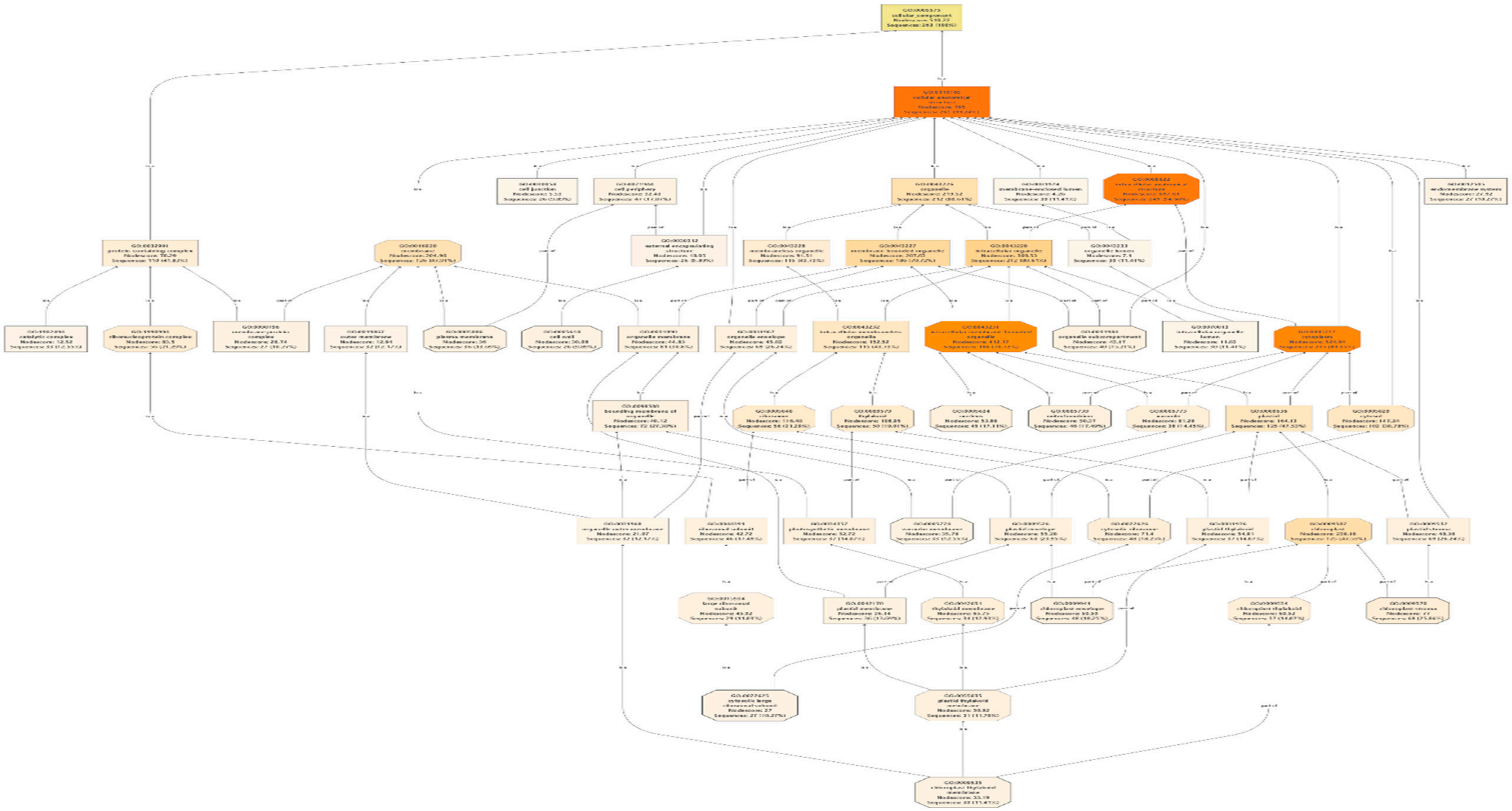

Supplementary Figure S4: Complete functional distribution of detected proteins from *Tetraselmis chuii* biomass in cellular component functional group using gene ontology (GO).
