# Supplementary material for: Tetraselmis chuii as Source of Bioactive Compounds Against Helicobacter pylori: An Integrated Proteomic and Bioactivity Approach
Source: Molecules. 2025 Dec 5;30(24):4669. doi: 10.3390/molecules30244669 (PMC12736364; doi:10.3390/molecules30244669)
Supplement: Supplementary file 1 [file molecules-30-04669-s001.zip › Supplementary Upload-Revision/molecules-3995090-supplementary-2.pdf]

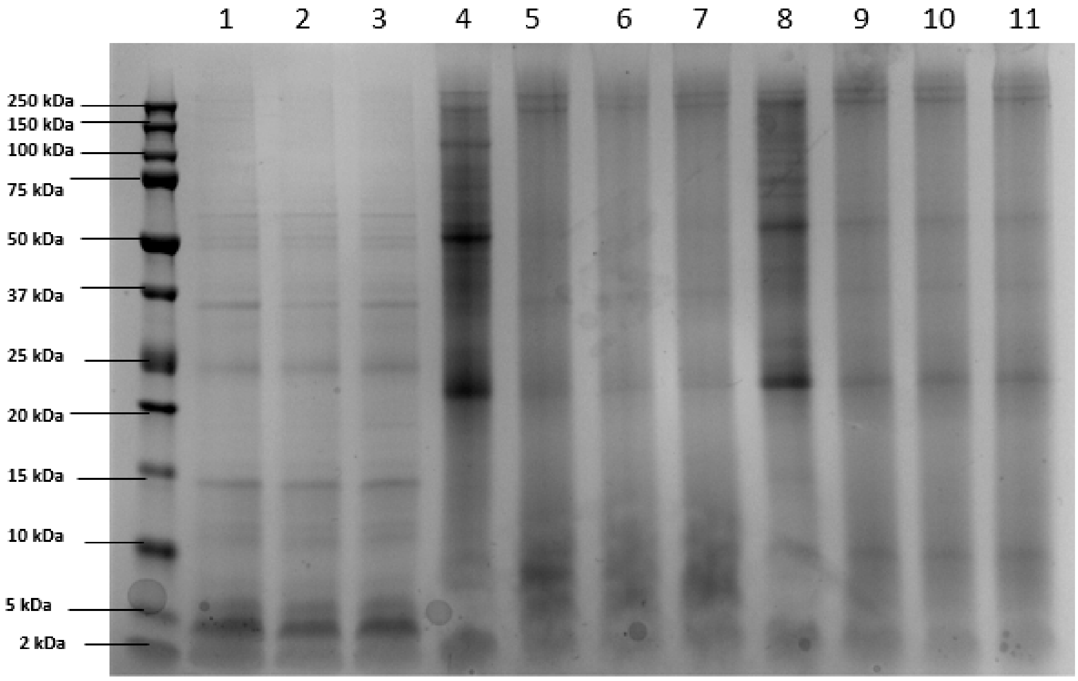

1. Blank R1
2. Blank R2
3. Blank R3
4. Untreated biomass *Tetraselmis*
5. Untreated and digested *Tetraselmis* R1
6. Untreated and digested *Tetraselmis* R2
7. Untreated and digested *Tetraselmis* R3
8. Treated (*C*/D + US) *Tetraselmis* Biomass
9. Treated and digested *Tetraselmis* R1
10. Treated and digested *Tetraselmis* R2
11. Treated and digested *Tetraselmis* R3

<u>Original image for figure 1</u>

Lanes 1-3, and 8-11 are the lanes that appear in the figure shown in the article.
